# EGR1 drives cell proliferation by directly stimulating TFEB transcription in response to starvation

**DOI:** 10.1371/journal.pbio.3002034

**Published:** 2023-03-08

**Authors:** Marcella Cesana, Gennaro Tufano, Francesco Panariello, Nicolina Zampelli, Susanna Ambrosio, Rossella De Cegli, Margherita Mutarelli, Lorenzo Vaccaro, Micheal J. Ziller, Davide Cacchiarelli, Diego L. Medina, Andrea Ballabio

**Affiliations:** 1 Telethon Institute of Genetics and Medicine (TIGEM), Pozzuoli, Naples, Italy; 2 Department of Advanced Biomedical Sciences, Federico II University, Naples, Italy; 3 Department of Biology, University of Naples ’Federico II’, Naples, Italy; 4 Istituto di Scienze Applicate e Sistemi Intelligenti “E. Caianiello,” Consiglio Nazionale Delle Ricerche, Pozzuoli, Italy; 5 Lab for Genomics of Complex Diseases, Max Planck Institute of Psychiatry, Munich, Germany; 6 Department of Psychiatry, University of Münster, Münster, Germany; 7 Department of Medical and Translational Science, Federico II University, Naples, Italy; 8 Department of Molecular and Human Genetics, Baylor College of Medicine, Houston, Texas, United States of America; 9 Jan and Dan Duncan Neurological Research Institute, Texas Children’s Hospital, Houston, Texas, United States of America; Consejo Nacional de Investigaciones Científicas y Técnicas: Consejo Nacional de Investigaciones Cientificas y Tecnicas, ARGENTINA

## Abstract

The stress-responsive transcription factor EB (TFEB) is a master controller of lysosomal biogenesis and autophagy and plays a major role in several cancer-associated diseases. TFEB is regulated at the posttranslational level by the nutrient-sensitive kinase complex mTORC1. However, little is known about the regulation of TFEB transcription. Here, through integrative genomic approaches, we identify the immediate-early gene EGR1 as a positive transcriptional regulator of TFEB expression in human cells and demonstrate that, in the absence of EGR1, TFEB-mediated transcriptional response to starvation is impaired. Remarkably, both genetic and pharmacological inhibition of EGR1, using the MEK1/2 inhibitor Trametinib, significantly reduced the proliferation of 2D and 3D cultures of cells displaying constitutive activation of TFEB, including those from a patient with Birt-Hogg-Dubé (BHD) syndrome, a TFEB-driven inherited cancer condition. Overall, we uncover an additional layer of TFEB regulation consisting in modulating its transcription via EGR1 and propose that interfering with the EGR1-TFEB axis may represent a therapeutic strategy to counteract constitutive TFEB activation in cancer-associated conditions.

## Abstract 2

The stress-responsive transcription factor TFEB is a master controller of lysosomal biogenesis and autophagy and plays a major role in several cancer-associated diseases. Integrative genomic analyses identify the immediate-early gene EGR1 as a positive transcriptional regulator of TFEB expression and reveal the function of the EGR1-TFEB transcriptional axis in physiology and disease.

## Introduction

Cells have evolved by improving their capacity to metabolically adapt to changes in substrate availability and energy requirements. This metabolic flexibility, essential to maintain energy homeostasis, is made possible by the coordinated interplay of diverse quality control mechanisms. In this scenario, transcriptional control of gene expression heavily impacts the homeostatic energy balance in both physiological and pathological conditions [1]. Importantly, acceleration of energy metabolism to fuel cell growth and division is a hallmark of cancer cells [2]. Indeed, cancer cells often exploit transcription factor-mediated catabolic programs to meet their requirements.

An example is transcription factor EB (TFEB), a member of the microphthalmia family (MiT-TFE) of bHLH-leucine zipper transcription factors, which act as a global modulator of intracellular clearance and energy metabolism through the control of lysosomal biogenesis and autophagy [3–5]. Besides its role in cellular metabolism, TFEB is a crucial player in cancer biology [6–8]. Recently, we and others showed that constitutive activation of TFEB, through the inhibition of the noncanonical mTORC1 pathway driven by FLCN-RagC/D, induces renal tumorigenesis in mouse models of Birt-Hogg-Dubé (BHD) syndrome [9,10] and tuberous sclerosis [11].

TFEB activity is known to be regulated at the posttranslational level through phosphorylation mediated by the nutrient-sensing complex mTORC1. Upon nutrient depletion, TFEB translocates to the nucleus and induces a global transcriptional response to ensure adaptation to changes in cells’ energy demands [5,12–15]. Despite the increasing recognition of TFEB’s role in controlling vital metabolic processes and its involvement in several diseases, a knowledge gap remains on the mechanisms and players controlling its transcriptional regulation and their relevance in cellular adaptation to environmental cues.

Here, we surveyed transcription factors for their capacity to control TFEB expression and identified the immediate-early gene EGR1 as a positive transcriptional regulator of TFEB. Stress signals and secreted factors, including growth factors, tumor necrosis factors, inflammatory factors, ionizing radiation, reactive oxygen species, or others, are known to trigger EGR1 transcription via the MEK1/2/ERK signaling cascade [16–20]. Once activated, EGR1 initiates a transcriptional cascade that impacts a remarkable spectrum of, often opposing, cellular mechanisms, including survival and apoptosis, growth control and arrest, differentiation, and transformation [21–23]. Here, we showed that EGR1 controls TFEB transcription and contributes to TFEB-driven starvation response, significantly impacting the expression of several cell cycle regulators. Finally, we provided evidence that genetic and pharmacological inhibition of EGR1 using the FDA-approved compound Trametinib impairs TFEB-driven cell proliferation. Collectively, our data identify a novel layer in the regulation of TFEB, which may be targeted for therapeutic purposes.

## Results

### Dissection of human TFEB locus revealed an elaborate structure with multiple regulatory regions

To dissect the transcriptional architecture of the human TFEB gene, we interrogated expression profile datasets of a wide range of human tissues of embryonic origin [24,25]. We observed that TFEB displays a ubiquitous expression with a median fluctuation within one order of magnitude between different tissues (**S1A Fig, left panel**). This trend is also maintained across several ENCODE reference cell lines [26], with human embryonic stem cells (hESCs) displaying the highest levels of TFEB expression (**S1A Fig, right panel**). Despite a widespread expression of TFEB across tissues, its genomic locus results in a complex exon–intron structure and chromatin makeup. **S1B Fig** shows the human TFEB locus with mRNA species harboring from the negative strand, transcribed from right to left. The TFEB gene contains alternative 5′ noncoding exons and an approximately 40-kb first intron. Analysis of TFEB transcripts revealed multiple splicing isoforms with a prevalence of isoforms containing 8 coding exons and a common 3′ UTR region (red), encoding a 476 amino acid protein. The primary reference transcriptional isoform (underlined) displays the first exon with a common expression in most of the ENCODE reference cell lines [26] (Transcription—highlighted with a light gray bar, arrow #1). To gain more insights into the structure of the TFEB locus, we performed a computational analysis integrating existing genomic and epigenetic data. Specifically, we leveraged H3K4me1, H3K4me3, and DNase I profiles from the ENCODE and Epigenome Roadmap Project [27] to locate putative *cis*-regulatory elements (CREs), which we numbered from 1 to 7 (**S1C Fig** and see Methods). In particular, CREs 1, 2, and 3 are enriched for the promoter-specific modification H3K4me3 and represent the region where most alternative transcriptional start sites are located (Promoters—H3K4me3). In particular, the strongest promoter signal common to the major reference cell lines is localized upstream of the major TSS (arrow #2), right next to the strongest chromatin accessibility mark in the region (arrow #3), suggesting histone displacement, TF engaging, and assembly of the transcriptional machinery.

The presence of an articulated, cell-specific H3K4me1 patterning in the TFEB locus suggests that distinct CREs are likely engaged in a cell-specific fashion (Enhancers—H3K4me1), as underlined by a different TFEB expression pattern across tissues (**S1A Fig**). Additionally, minor alternative TSS arising from CREs with a little promoter activity (i.e., 5 and 7) generate low abundant mRNAs without different protein-coding features compared to the major isoform. The engagement of differential CREs, labeled by enhancers, promoters, and/or accessibility marks, is further confirmed by chromatin folding data (Hi-C), which displays proximity contacts between the promoter regions and the other CREs enriched for the H3K4me1 enhancer mark (**S1D Fig**). Lastly, a long-distance interaction was observed between the promoter and the transcriptional termination region, with a concomitant “carry-over” of promoters and chromatin accessibility marks (arrow #4), as previously reported for actively transcribed loci [28]. In line with this observation, hESCs display the highest degree of enrichment of H3K4me1 and DNAseI accessibility in this region, thus resulting in a high level of TFEB expression (**S1A Fig, right panel**).

### EGR1 regulates TFEB transcription

**Fig 1:**
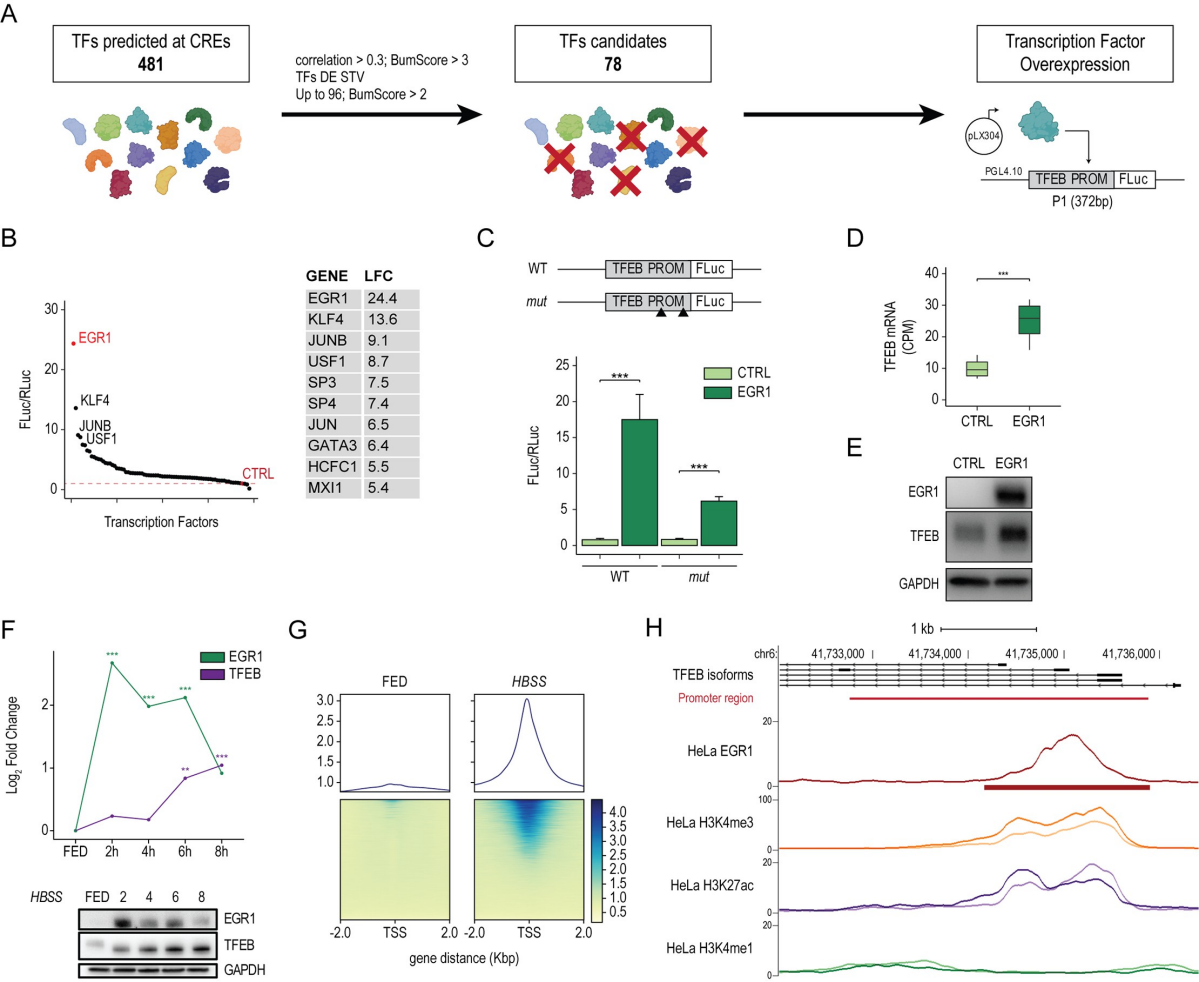
EGR1 positively modulates TFEB transcription. **(A)** Schematic representation of the filtering strategy to identify regulators of TFEB transcription. TFs were selected according to several criteria: (i) the number of predicted binding sites at the level of the identified CREs (Bumscore >3) and their correlation of expression with TFEB (Pearson correlation >0.3); (ii) TFs differentially expressed upon starvation (TFs DE STV); (iii) TFs with a Bumscore >2 up to 96 TFs. Selected TFs were cloned in the pLX304 vector and overexpressed in HeLa cells along with the TFEB promoter-reporter (TFEB PROM), in which the TFEB promoter region was cloned upstream of the firefly luciferase reporter gene (FLuc). (**B)** Survey of candidate TFs through luciferase-based promoter assay. (Left) Dot plot showing the luciferase activity relative to each TF measured as FLuc/RLuc ratio shown with respect to the empty control vector (CTRL). (Right) Table of the top 10 TFs with corresponding Log FC values (LFC) relative to the ratio FLuc/RLuc. (**C**) TFEB promoter activity assay. TFEB promoter (WT) and its mutated version for EGR1 binding sites (*mut*) were cloned upstream of the firefly luciferase coding region (FLuc). These constructs were cotransfected in HeLa cells alongside plasmids expressing EGR1 open reading frame or with a control empty vector (CTRL). A construct carrying the Renilla luciferase (RLuc) was transfected as a control. Bar plot showing the luciferase activity measured as FLuc/RLuc ratio shown with respect to the control vector. Mean ± SD values are shown. ANOVA was used; *p < 0.05, **p < 0.01, ***p < 0.001. (**D)** Normalized expression (CPM) of TFEB mRNA in HeLa cells upon CTRL and EGR1 overexpression measured by RNAseq (*** FDR < 0.001). (**E)** Representative image of the immunoblot analysis of TFEB and EGR1 levels upon EGR1 overexpression in HeLa cells (*n =* 3). GAPDH was used as a loading control. (**F)** (Upper) Line plot showing EGR1 and TFEB mRNA dynamics during a starvation time course. HeLa cells were treated with HBSS for the indicated time points. EGR1 and TFEB mRNA levels were quantified by RNAseq and shown as Log_2_FC with respect to their levels in FED conditions (** FDR < 0.01; *** FDR < 0.001). (Lower) Representative image of immunoblot analysis of EGR1 and TFEB levels in HeLa cells treated with HBSS for the indicated time points. GAPDH was used as a loading control. (**G)** ChIPseq analysis of HeLa cells undergoing starvation (HBSS) for 6 hours compared to cells in fed condition (FED). (Upper) Distribution plots of the average read coverage density of EGR1 binding signal within 2 kb from all TSSs in the genome. (Lower) Binding heatmaps displaying the individual read coverage density of the EGR1 binding signal. (**H)** Representative genome browser snapshots of TFEB promoter bound by EGR1 in starvation. Both reads distributions as line plots and peak intervals are displayed. H3k4me3, H3k4me1, and H3K27ac enrichments at the TFEB promoter during starvation are also displayed. The genomic localization of the TFEB promoter region (as described in S1 Fig) is reported (red line). Individual quantitative observations that underlie the data summarized here can be located under the Supporting information file as S1 Data. Uncropped images can be found in the Supporting information file as S1 Raw Images. ChIPseq, chromatin immunoprecipitation sequencing; CRE, *cis*-regulatory element; CTRL, control; EGR1, early growth response 1; FLuc, firefly luciferase; LFC, Log FC; RLuc, Renilla luciferase; TF, transcription factor; TFEB, transcription factor EB; TSS, transcriptional start site; WT, wild-type.

To identify bona fide TFEB transcriptional regulators, we selected 78 TFs predicted to bind to multiple sites in the CREs 1 to 7 regions of the TFEB locus and whose expression showed higher correlation scores with TFEB expression (see Methods) (**Fig 1A)**. We identified CRE1 as the core-promoter region of TFEB (TFEB PROM), as it resides immediately upstream of the major TSS (arrow #1) and exhibits the highest levels of histone displacement (arrow #2) and chromatin accessibility (arrow #3). Plasmids containing the selected TFs were cotransfected into HeLa cells together with a reporter construct in which the TFEB promoter region was fused with the firefly luciferase coding region.

This analysis revealed that 10 TFs could induce TFEB promoter activity above 5-fold compared to the control. Among them, the early growth response 1 (EGR1) TF displayed the strongest ability to increase the luciferase activity driven by the TFEB promoter and was chosen for further analyses (**Fig 1B**). EGR1 belongs to the immediate-early genes (IEGs) family and represents the earliest downstream nuclear target sensitive to changes in the extracellular environment, including nutrients and stress signals [29], which triggers its activation via the MEK1/2/ERK signaling pathway [21,30]. Therefore, based on the established role of TFEB as a nutrient and stress sensor in the cell, we hypothesized a functional correlation between EGR1 and TFEB.

To validate EGR1 binding to the TFEB promoter, we cotransfected TFEB PROM and plasmids for EGR1 overexpression (EGR1) or control (CTRL) into HeLa cells. We observed a significant induction of luciferase activity relative to the wild-type (WT) promoter. Conversely, luciferase activity was significantly reduced in the presence of a TFEB PROM in which EGR1 binding sites were mutated (*mut*) (**Fig 1C**). To further confirm the finding that EGR1 positively controls TFEB expression, we overexpressed EGR1 in HeLa cells and performed RNAseq. We observed that TFEB mRNA and protein levels were significantly induced by EGR1 (**Fig 1D and 1E**). Gene ontology (GO) enrichment analysis of genes up-regulated upon EGR1 overexpression using both manually curated gene sets and KEGG pathways highlighted TFEB-regulated pathways, including “Autophagy,” “Lysosome” [5], and “mTOR signaling,” along with others, such as “TNF signaling,” “Epithelial-Mesenchymal Transition,” and “Hypoxia” (**S2A Fig**). In line with the GO results, we observed an induction of TFEB targets belonging to several pathways, including autophagy genes, lysosomal genes, and components of the mTORC1 signaling pathway (**S2B Fig**). Collectively, these results demonstrate that EGR1 regulates TFEB expression and its downstream transcriptional network.

### EGR1 associates with TFEB promoter upon starvation

Upon starvation, TFEB rapidly translocates to the nucleus and activates the transcription of its target genes [15]. As EGR1 is activated upon various stimuli, including nutrient depletion [21], we sought to investigate whether it contributes to the nutrient-sensing response by regulating TFEB transcription. Thus, we performed a starvation time course and analyzed the expression dynamics of both EGR1 and TFEB by RNAseq. EGR1 mRNA levels are low in basal conditions (FED) and significantly increase from 2 hours of starvation (2h) to remain high until 6 hours, after which they start decreasing but still remain higher than in the basal condition. Differently, TFEB expression starts to significantly increase from 6 hours of starvation (6h), reaching its maximum at 8 hours (8h) (**Fig 1F, upper panel**). Analysis of EGR1 and TFEB protein levels by western blot reflected their transcriptional dynamics (**Fig 1F, lower panel**). To test whether a functional correlation exists between EGR1 and TFEB during starvation, we evaluated endogenous EGR1 occupancy genome-wide by performing chromatin immunoprecipitation sequencing (ChIPseq) in HeLa cells in fed and starved conditions (HBSS). ChIPseq analysis revealed that, upon starvation, EGR1 associates with its target genes (**Fig 1G**) and is preferentially enriched at the TFEB promoter region, marked by the histone marker H3K4me3 (**Fig 1H**), supporting the hypothesis that EGR1 is responsible for TFEB mRNA induction during nutrient depletion. We also evaluated whether genes bound by EGR1 were up-regulated during starvation. Surprisingly, we observed that only 4.3% of them were up-regulated, whereas, for most of them, the expression was unchanged or reduced (**S3A Fig**). Globally, GO analysis of EGR1 targets up-regulated in starvation highlighted, among others, categories related to “TNF-alpha signaling,” “Hypoxia,” “Mitophagy,” and “G2-M checkpoint” (**S3B Fig**). Collectively, these data demonstrate that EGR1 association to the chromatin is triggered by starvation and that TFEB is among those few EGR1 targets to be up-regulated, further emphasizing a functional correlation between EGR1 and TFEB.

### EGR1 depletion dampens TFEB-mediated transcriptional response to starvation

**Fig 2:**
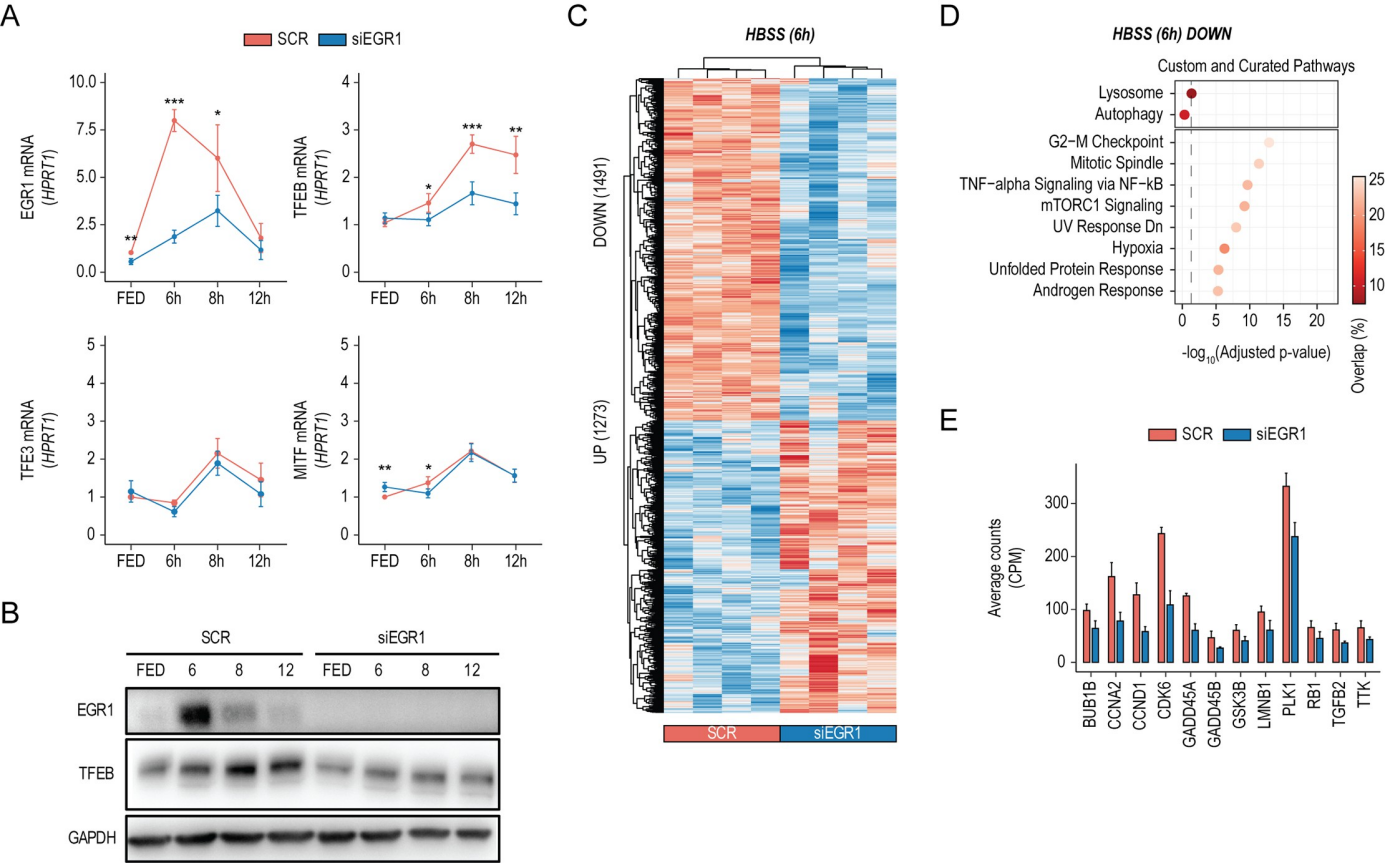
EGR1 depletion dampens TFEB-mediated transcriptional response to starvation. **(A)** Line plots showing relative EGR1, TFEB, TFE3, and MITF mRNA levels measured by qPCR in HeLa cells treated with siRNAs targeting EGR1 (siEGR1) or scramble sequences (SCR) in fed (FED) and starved conditions for the indicated time points. Values were normalized on the HPRT expression and displayed as a fold change relative to scramble-treated samples in FED conditions set to 1. Mean ± SD values are shown. ANOVA was used; *p < 0.05, **p < 0.01, ***p < 0.001. (**B)** Immunoblot analysis of EGR1 and TFEB expression in HeLa cells treated with siRNAs targeting EGR1 (siEGR1) or scramble sequences (SCR) in FED conditions or during starvation for the indicated time points. GAPDH was used as a loading control. (**C)** Heatmap of Z-scored log_2−_normalized expression values of deregulated genes (UP: 1,273, DOWN: 1,491) upon siEGR1 in HeLa cells after 6 hours of starvation (HBSS 6h). Replicates for SCR and siEGR1 conditions are reported. (**D)** Balloon plots of representative term enrichment analysis results using Custom Genesets (Autophagy and Lysosome) and Curated Pathways (KEGG and MSigDB Hallmark collection) of down-regulated genes upon siEGR1 treatment, with respect to scramble-treated cells. Enriched terms are ranked by adjusted *p*-value (x-axis), and the balloon color scale represents the percentage of overlap between the input genes and the analyzed term. Significance threshold (dashed line, adjusted *p*-value < 0.05) is reported. (**E)** RNAseq-based expression (CPM) of representative genes down-regulated upon siEGR1 in HeLa. Mean ± SD values are shown. Individual quantitative observations that underlie the data summarized here can be located under the Supporting information file as S1 Data. Uncropped images can be found in the Supporting information file as S1 Raw Images. EGR1, early growth response 1; TFEB, transcription factor EB.

**Fig 3:**
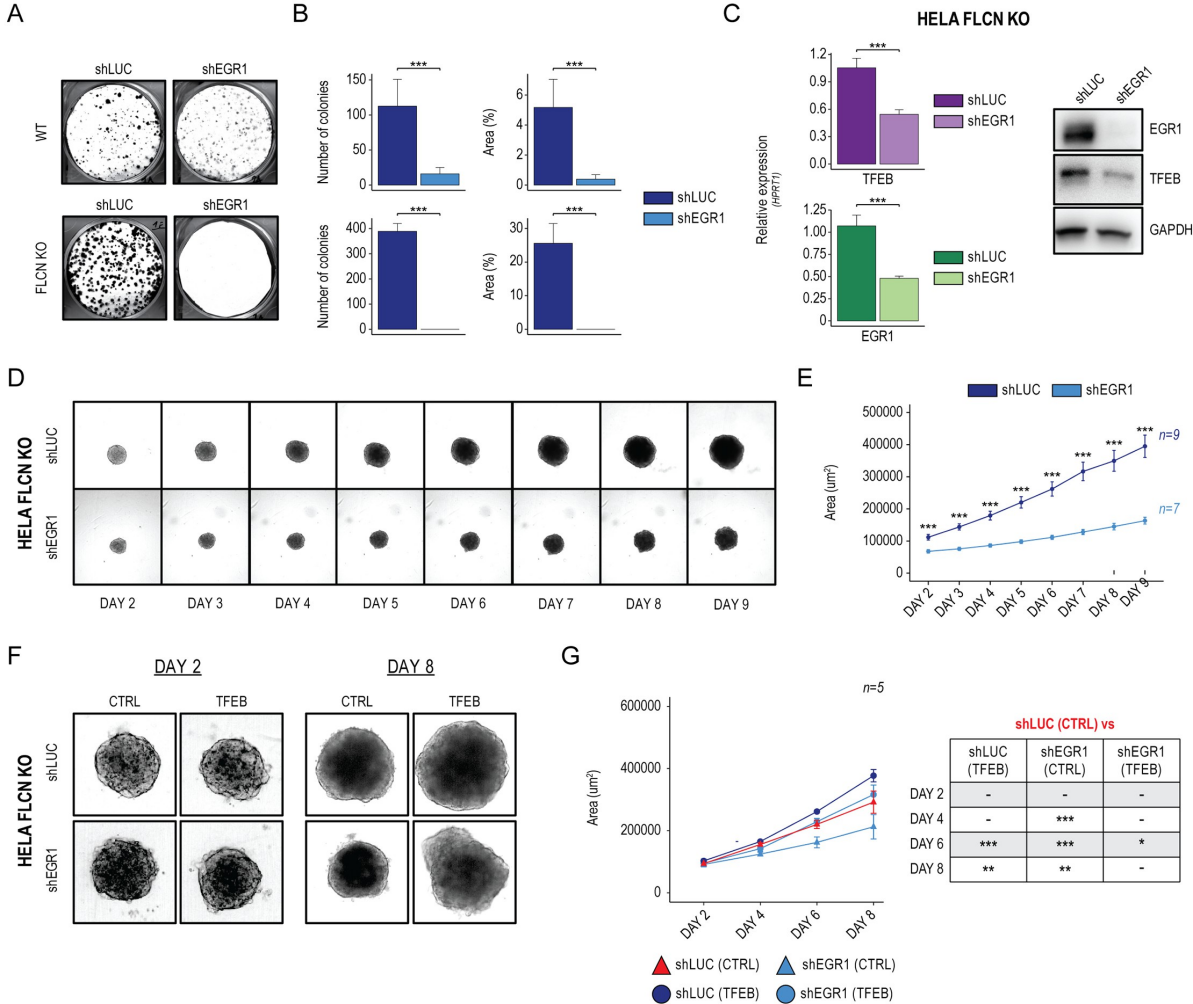
Modulation of EGR1 impacts on TFEB-driven tumorigenesis. **(A)** Representative image of colony formation capacity of HeLa WT (upper) and HeLa-FLCN KO cells (lower) stably infected with shRNAs against luciferase (shLUC) and EGR1 (shEGR1). (**B)** Bar plots showing the quantification of colonies’ number and size (%Area) in shLUC and shEGR1 engineered cells. Mean ± SD values are shown. ANOVA was used; *p < 0.05, **p < 0.01, ***p < 0.001. (**C)** (Left) Bar plots showing TFEB and EGR1 mRNAs quantification as measured by qPCR in HeLa-FLCN KO cells treated with shLUC and shEGR1. Mean ± SD values are shown. ANOVA was used; *p < 0.05, **p < 0.01, ***p < 0.001. (Right) Representative image of immunoblot analysis of EGR1 and TFEB levels in HeLa-FLCN KO cells stably infected with shLUC and shEGR1. GAPDH was used as a loading control. (**D)** Bright-field representative images of 3D spheroids generated from HeLa-FLCN KO cells stably infected with shLUC and shEGR1 over a 9-day course. (**E)** Line plot showing High-Content Imaging-based quantification of 3D spheroids size (Area µm^2^). Mean ± SD values relative to 9 and 7 replicates for shLUC and shEGR1 spheroids, respectively. ANOVA was used; *p < 0.05, **p < 0.01, ***p < 0.001. (**F)** Bright-field representative images of 3D spheroids generated from shLUC and shEGR1 HeLa-FLCN KO cells infected with a doxycycline-inducible TFEB overexpressing construct. Images are relative to 3D spheroids at 2 and 8 days of culture, in the absence (CTRL) or presence (TFEB) of doxycycline. (**G)** (Left) Line plot showing High-Content Imaging-based quantification of 3D spheroids size (Area µm^2^) over 8 days of culture, in the absence (CTRL) or presence (TFEB) of doxycycline. Mean ± SD values relative to 5 replicates for each condition. (Right) Results from the ANOVA followed by Tukey’s multiple comparisons significance test; *p < 0.05, **p < 0.01, ***p < 0.001. Individual quantitative observations that underlie the data summarized here can be located under the Supporting information file as S1 Data. Uncropped images can be found in the Supporting information file as S1 Raw Images. EGR1, early growth response 1; TFEB, transcription factor EB; WT, wild-type.

To evaluate the effect of EGR1 down-regulation upon nutrient depletion, we performed a starvation time course in cells in which EGR1 expression was silenced using siRNAs (siEGR1). EGR1 silencing significantly dampened the starvation-induced TFEB increase observed in the control sample starting from 6 hours of treatment (**Fig 2A**). In line with this finding, immunoblot analysis revealed a reduction of TFEB protein levels in siEGR1-treated cells (**Fig 2B**). Conversely, the expression levels of TFE3 and MITF, other members of the MiT/TFE family of transcription factors, were not altered by the down-regulation of EGR1 during starvation (**Fig 3A**), suggesting that EGR1 exerts a transcriptional control on TFEB only.

To evaluate the effect of EGR1 down-regulation on the transcriptome, we performed RNAseq analysis of EGR1-silenced HeLa cells undergoing starvation for 6 hours (**Fig 2C**). In line with EGR1-mediated down-regulation of TFEB, “Autophagy” and “Lysosome” pathways were enriched for down-regulated genes (**Fig 2D**).

Interestingly, cell cycle–related pathways (i.e., “G2-M Checkpoint” and “Mitotic spindle”) scored as the most significant GO terms enriched for genes affected by EGR1 down-regulation. Indeed, we identified several crucial cell cycle regulators significantly down-regulated upon siEGR1 treatment (**Fig 2E**). Together, our data indicate that EGR1 depletion hampers TFEB transcriptional induction upon starvation, impacting TFEB-mediated gene networks.

### Modulation of EGR1 impacts TFEB-driven cell proliferation

TFEB has recently emerged as a critical player in a wide array of cancer-associated conditions, in which it was found to promote tumorigenesis [6]. Indeed, in a previous study, we showed that renal tumorigenesis in BHD syndrome is caused by constitutive TFEB activation due to FLCN loss of function [9]. Thus, we sought to evaluate whether modulation of EGR1 expression influences the oncogenic properties of constitutive TFEB activation. We measured the clonogenic capacity of cells stably expressing short-hairpin RNAs against EGR1 (shEGR1) through a colony formation assay (CFU). HeLa WT cells treated with shEGR1 significantly reduced the number and size of the colonies compared to the control (shLUC) (**Fig 3A**, upper panel). This effect was significantly enhanced in HeLa cells depleted for FLCN (HeLa-FLCN KO), where TFEB is constantly nuclear, and acting on its expression levels might represent a valuable strategy to reduce its activity. Indeed, we observed a complete abolishment of colonies output in shEGR1-treated cells. Notably, in line with the oncogenic role of a constitutively active TFEB, we observed that HeLa-FLCN KO cells form more colonies than HeLa WT cells (**Fig 3A**, lower panel). Quantification of both colony number and size confirmed that shRNA-mediated down-regulation of EGR1 impacts the clonogenic capacity of HeLa WT and, more pronouncedly, HeLa-FLCN KO cells (**Fig 3B**). Measurement of mRNA levels by quantitative RT-PCR (**Fig 3C**, left panel) and immunoblot analysis (**Fig 3C**, right panel) confirmed a significant decrease of TFEB expression upon shEGR1 in HeLa-FLCN KO.

To further investigate to which extent reduced EGR1 levels affect the oncogenic properties of HeLa-FLCN KO cells, we developed a 3D cellular system (3D spheroids) that better mimics the structural organization of a solid tumor (**Fig 3D**). Over 9 days, we monitored 3D spheroids’ growth in culture and quantified their size by High-Content Imaging. 3D spheroids derived from HeLa-FLCN KO infected with shEGR1 displayed a significant reduction in the kinetic of growth compared to control-treated cells (shLUC) (**Fig 3E**).

To prove that the impairment of shEGR1-derived spheroids’ growth was due to EGR1-mediated down-regulation of TFEB, we transduced shEGR1-treated cells with a lentiviral construct for TFEB overexpression (**Fig 3F**). Quantification of spheroids’ size showed that TFEB overexpression could rescue the growth defects of shEGR1-derived spheroids (**Fig 3G**). Interestingly, in line with previous observations suggesting the involvement of TFEB in cell migration [31–33], we observed a significant increase in the migration capacity of HeLa-FLCN KO overexpressing TFEB, strengthening TFEB’s oncogenic role (**S4A and S4B Fig**).

Collectively, these data indicate that HeLa-FLCN KO cells are sensitive to EGR1 down-regulation, which affects their oncogenic features by reducing TFEB expression.

### Trametinib inhibits the EGR1-TFEB axis

**Fig 4:**
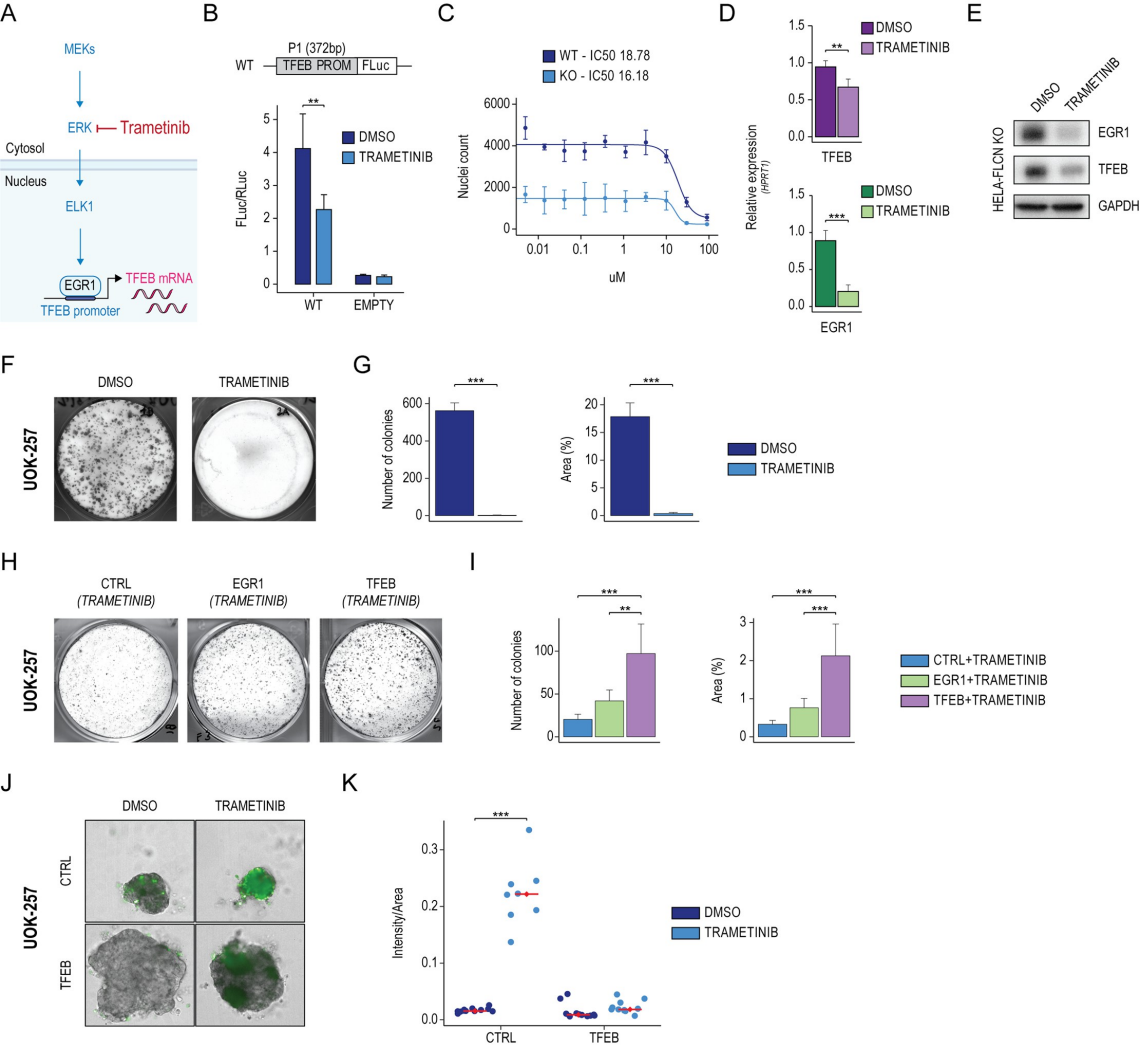
Trametinib inhibits the EGR1-TFEB axis in BHD patient-derived cancer cells. **(A)** Schematic representation of the MEK1/2/ERK signaling cascade, which regulates EGR1 activation. (**B)** TFEB promoter activity assay upon Trametinib treatment. TFEB promoter (WT), along with a control vector (EMPTY), were transfected in HeLa cells for 48 hours, following starvation (HBSS) for 6 hours in the presence of DMSO or Trametinib. Bar plot showing the luciferase activity measured as FLuc/RLuc ratio. Mean ± SD values are shown. ANOVA was used; *p < 0.05, **p < 0.01, ***p < 0.001. (**C)** Trametinib dose–response curve. HeLa WT and HeLa-FLCN KO (KO) cells were treated with serial dilutions of Trametinib. The half maximal inhibitory concentration (IC50) was calculated by counting the nuclei of viable cells. (**D)** Bar plots showing relative quantification of EGR1 and TFEB mRNA levels measured by qPCR in HeLa-FLCN KO cells treated with DMSO or Trametinib. Values were normalized on the HPRT expression and displayed as a fold change relative to DMSO. Mean ± SD values are shown. ANOVA was used; *p < 0.05, **p < 0.01, ***p < 0.001. (**E)** Immunoblot analysis of EGR1 and TFEB expression in HeLa-FLCN KO cells treated with DMSO and Trametinib. GAPDH was used as a loading control. (**F)** Representative image of colony formation capacity of UOK-257 cells treated with DMSO and Trametinib. (**G)** Bar plots showing the quantification of colonies’ number and size (%Area) relative to indicated treatments. Mean ± SD values are shown. ANOVA was used; *p < 0.05, **p < 0.01, ***p < 0.001. (**H)** Representative image of colony formation capacity of UOK-257 cells transduced with control (CTRL), EGR1, and TFEB overexpression vectors and treated with Trametinib. (**I)** Bar plots showing the quantification of colonies’ number and size (%Area) relative to indicated treatments. Mean ± SD values are shown. ANOVA followed by Tukey’s multiple comparisons test was used; *p < 0.05, **p < 0.01, ***p < 0.001. (**J)** Bright-field and fluorescence representative images of 3D spheroids generated from UOK-257 cells transduced with control (CTRL) and TFEB overexpression vectors and treated with DMSO and Trametinib. (**K)** Dot plot showing the ratio of the intensity of the cell death dye over the size of each spheroid (Intensity/Area) derived from CTRL and TFEB-engineered UOK-257 cells upon indicated treatments. Mean ± SD values are shown. ANOVA was used; *p < 0.05, **p < 0.01, ***p < 0.001. Individual quantitative observations that underlie the data summarized here can be located under the Supporting information file as S1 Data. Uncropped images can be found in the Supporting information file as S1 Raw Images. BHD, Birt-Hogg-Dubé; CTRL, control; EGR1, early growth response 1; TFEB, transcription factor EB; WT, wild-type.

The mitogen-activated protein kinase (MAPK) signaling pathway is a well-established positive regulator of EGR1 expression [34]. Recently, the MEK1/2 inhibitor Trametinib, an FDA-approved compound, was shown to suppress inflammation in LPS-activated macrophages by inhibiting the MEK-ERK-Egr-1 pathway [35] (**Fig 4A**). Therefore, we test whether Trametinib affected TFEB transcription through inhibition of EGR1 and transfected the TFEB promoter construct (WT) in HeLa cells in the presence of Trametinib or DMSO as control. Luciferase assay showed that Trametinib reduced the activity of the TFEB promoter, proving that Trametinib negatively impacts TFEB transcription (**Fig 4B**). In accordance with the notion that EGR1 is a well-established target of the MEK-ERK pathway [30,35], we showed that the increase of EGR1 expression observed in starvation correlated with the activation of the MEK-ERK pathway, measured by the increase of the phosphorylation state of ERK by immunoblot analysis. Notably, Trametinib treatment completely abrogates pERK and, consequently, EGR1 expression, suggesting that EGR1 activation upon starvation depends on MERK-ERK pathway activation (**S5A and S5B Fig**).

In line with this, the efficacy of Trametinib treatment in reducing cell viability (IC50 of 16.18 µM in HeLa-FLCN KO versus 18.78 µM in WT cells) was more pronounced in HeLa FLCN-KO cells, where TFEB is active (**Fig 4C**). On a genome-wide level, Trametinib treatment in HeLa-FLCN KO cells negatively affected the expression of genes belonging to cell cycle–related pathways (i.e., “G2-M Checkpoint” and “Mitotic Spindle”) (**S5C and S5D Fig**). In agreement with the luciferase assay results, Trametinib significantly reduced endogenous TFEB and EGR1 at both RNA (**Fig 4D**) and protein levels (**Fig 4E**) in HeLa-FLCN KO cells.

### Trametinib inhibits cell proliferation in BHD patient-derived cancer cells

To analyze the effect of Trametinib on cell proliferation, we treated the UOK-257 cell line derived from a BHD patient [36] and measured its clonogenic capacity (**Fig 4F**). Quantification of both colony number and size revealed that Trametinib impacts the clonogenic capacity of UOK-257 cells (**Fig 4G**). EGR1 and TFEB overexpression in Trametinib-treated UOK-257 cells increased both colony number and size compared to the control, thus dampening the effect of Trametinib (**Fig 4H and 4I**). Next, we developed 3D spheroids from CTRL- and TFEB-engineered UOK-257 cells and found that the area of 3D spheroids overexpressing TFEB significantly increased compared to the control, suggesting an increase in cell growth and proliferation (**Fig 4J**).

Remarkably, High-Content Imaging-based evaluation of cell viability showed that TFEB overexpressing cells are more resistant to Trametinib treatment (**Fig 4K**). Together, these data indicate that Trametinib affects the proliferation of BHD patient-derived cancer cells by acting on the EGR1-TFEB axis.

## Discussion

Lack of in-depth knowledge of how TFEB is regulated at the transcriptional level has hindered a complete understanding of the mechanisms that control the TFEB-mediated response to environmental cues and metabolic needs. Indeed, TFEB was shown to be controlled at the posttranslational level through the regulation of its phosphorylation state by mTORC1, which dictates its subcellular localization [5,12,13,15]. However, transcriptional regulation represents an additional important layer to control TFEB activity, with significant implications for cellular metabolism. Indeed, multiple studies correlate increased levels of TFEB with poor prognosis in several types of cancers, including glioblastoma [37], non-small lung cancer [38], breast carcinoma [39], and prostate cancer [40]. Recently, we demonstrated that sustained activation of TFEB is the main driver of renal tumorigenesis in a mouse model of BHD syndrome, which is caused by FLCN mutations, prompting research into strategies to counteract TFEB activation [9].

Here, we identified the IEG EGR1 as a modulator of TFEB transcription during starvation. By extensively dissecting the architecture of the human TFEB locus, we demonstrated that EGR1 specifically associates with the TFEB promoter to modulate its transcription and consequent activation of the downstream transcriptional network during starvation. Interestingly, evaluation of the transcriptional outputs upon EGR1 down-regulation highlighted that the EGR1-TFEB axis mainly impinges on the proliferative capacity of cells by affecting the expression of genes belonging to cell cycle–related pathways. Future analyses of genes significantly regulated by the EGR1-TFEB axis will be required to determine direct targets and comprehensively delineate the molecular pathways underlying TFEB-driven proliferation. A relevant consequence of our findings is the possibility of targeting EGR1 to modulate TFEB-driven functions in those conditions where its aberrant activation is detrimental. Indeed, we demonstrated that genetic down-regulation of EGR1 in HeLa cells depleted for FLCN, as a cellular model of TFEB-driven tumorigenesis, significantly reduced TFEB levels and, more importantly, inhibited the clonogenic capacity of these cells.

Interestingly, HeLa-FLCN KO cells are more sensitive to EGR1 down-regulation than WT cells. Indeed, in pathological conditions where TFEB is constitutively active due to inhibition of its canonical posttranslational regulation mechanism, interfering with its transcription may be an effective therapeutic strategy to decrease TFEB activity. To further confirm the relevance of modulating EGR1 to reduce TFEB-driven oncogenic properties, we developed 3D cultures from HeLa-FLCN KO cells to mimic the tumor microenvironment better. Using this model system, we demonstrated significant inhibition of TFEB-mediated induction of cell proliferation upon EGR1 down-regulation.

Recent studies have shown that EGR1 is closely related to the initiation and progression of cancer and may participate in tumor cell proliferation, invasion, metastasis, and tumor angiogenesis [41,42]. In particular, high EGR1 levels were detected in prostate cancer [43]. Furthermore, EGR1 has been implicated as an important factor in nephrogenesis and the development of renal cancer [44,45]. Future studies will determine whether EGR1-mediated activation of TFEB may be linked to tumorigenesis in EGR1-driven tumors. On the other hand, as overexpression of MITF/TFE family members is responsible for several types of cancer, including kidney cancer, it would be worthwhile to study the contribution of EGR1 to these cancers in vivo.

The knowledge that EGR1 expression is regulated by the MAPK signaling pathway [34] prompted us to employ Trametinib, the MEK1/2 inhibitor FDA-approved compound for melanoma, as a tool to reduce EGR1-TFEB expression in TFEB-driven cancers. We proved that Trametinib effectively abolished the clonogenic potential of cellular models of BHD syndrome. Importantly, overexpression of TFEB in Trametinib-treated UOK-257 cells, derived from a kidney tumor of a patient with BHD syndrome, resulted in a significant recovery of the clonogenic potential in 2D cultures and an increased resistance to cell death in 3D spheroids, suggesting that the Trametinib effect is mediated, at least in part, by modulation of TFEB levels.

In conclusion, our findings dissected a novel and relevant aspect of the regulation of TFEB activity and suggest that targeting TFEB transcription may represent an effective strategy to tackle conditions characterized by detrimental TFEB hyperactivation.

## Material and methods

### Cell culture

All cells were cultured in DMEM-High glucose (cat. no. ECM0728L, Euroclone) supplemented with 10% FBS (cat. no. ECS0180L, Euroclone), penicillin (100 IU/ml), and streptomycin (100 μg/ml) (cat. no. ECB3001D, Euroclone). For the starvation treatments, cells were washed twice using PBS Ca^2+^/Mg^++^ free, and HBSS (14025092, Gibco) supplemented with HEPES (H0887, Euroclone) was added for the indicated time points.

HeLa cells were obtained from ATCC. HeLa-FLCN KO cells were a kind gift of Z. P. Arany. UOK-257 cells were obtained from W. Marston Linehan, MD (The National Cancer Institute, Bethesda). All cell culture incubations were performed at 37 degrees with 5% CO_2_.

cDNA sequences for overexpression of EGR1 and TFEB were cloned into pLVX-EF1a-PURO and pLVX-TetOne-Puro (Clontech) lentiviral backbones, respectively. For RNA interference, shRNA sequences against EGR1 were cloned in the pLKO.1_hPGK-Puro-CMV-tGFP lentiviral backbone (Sigma-Aldrich).

### Viral transduction

Lenti-X 293T cell line (Takara) was used to produce lentiviral particles using standard procedures [46]. For viral transduction, cells were seeded at a density of 5 × 10^4^ cells per well in a 24-well plate, and viral particles were added at a multiplicity of infection (MOI) of 2 to 5 in a final volume of 500 µL in the presence of polybrene at 8 µg/mL final concentration. Media was changed 24 hours postinfection, and cells were selected using puromycin at a final concentration of 1 µg/mL.

### Prediction of TFEB transcriptional regulators

To identify transcription factors that regulate the expression of TFEB, we performed an integrated computational analysis that takes advantage of large existing gene expression resources and epigenetic data. In particular, we identified all putative enhancer regions within 20 kb of the TSS of the TFEB locus based on H3K27ac peak data across 90 distinct cell types and tissues from the ENCODE and Epigenome Roadmap Project. Subsequently, we pinpointed all transcription factors predicted to bind these enhancer regions based on the underlying enhancer DNA sequence to identify candidates potentially regulating TFEB. Next, we correlated the expression of these candidate transcription factors with the H3K27ac enrichment at the identified enhancer regions. Finally, we compiled a ranked list based on a computed score based on TFEB coexpression, promoter accessibility, and the number of predicted binding sites.

### Luciferase reporter assay

For the WT version, the TFEB promoter sequence was cloned into PGL4.10 plasmid (Promega) downstream of the firefly luciferase (FLuc) ORF. A mutant derivative mutated for predicted EGR1 binding sites (mut) was synthesized (GeneScript). A plasmid encoding for the Renilla luciferase gene (pRL-TK, Promega) was cotransfected to normalize for transfection efficiency. PGL4.10 empty plasmid was used as a negative control. FLuc and RLuc activities relative to WT and mut constructs were measured by Dual-Luciferase assay (Promega) 72 hours after transfection, and ratios between FLuc and RLuc were calculated.

### Western blot

Total cell extracts were obtained using SDS Lysis Buffer (10 mM Tris–HCl (pH 8), 0.2% SDS) with the addition of protease inhibitors (Sigma Aldrich, P8340) and sonicated briefly with Bioruptor. Protein concentration was measured by BCA assay (Pierce BCA Protein Assay Kit). Then, 15 µg of lysate was used for western blotting.

The proteins are resolved on precast gradient gel 4% to 12% (Invitrogen, NuPAGE 4% to 12%, Bis–Tris, 1.0 to 1.5 mm, Mini Protein Gels, cat# NP0323PK2) and then transferred to PVDF membrane.

PVDF membranes were blocked with 5% milk in TBST 0.1% for 1 hour at RT. Primary antibodies (in TBST 0.1% BSA 3%) were incubated overnight at 4°C. Membranes were incubated for 1 hour at RT with HRP-conjugated secondary antibodies (TBST 0.1% milk 5%). Each step was interspersed with the washing procedure in TBST 0.1%. Images were digitally acquired using an ImageQuant LAS4000 (GE Healthcare).

### RNA extraction, cDNA synthesis, and quantitative PCR

Total RNA was isolated using the RNeasy Plus mini kit (Qiagen) according to the manufacturer’s instructions. cDNA was synthesized using the QuantiTect Reverse Transcription kit (Qiagen). qPCR was performed with the LightCycler 480 SYBR Green I mix (Roche) using the LightCycler 480 II detection system (Roche). QuantiTect Primer Assays from Qiagen were used to quantify genes of interest. HPRT1 (Hs_HPRT1_1_SG QuantiTect Primer Assay, Qiagen, QT00059066) was used as an endogenous housekeeping control, and results were displayed as fold change compared to control samples. The following probes were used to quantify TFEB and EGR1: Hs_TFEB_1_SG QuantiTect Primer Assay (Qiagen, QT00069951) and Hs_EGR1_1_SG QuantiTect Primer Assay (Qiagen, QT002185051).

### siRNA transfection

Transfection of siRNAs was performed in suspension using Lipofectamine RNAiMAX Transfection Reagent (no. 13778, Invitrogen). Human siRNA EGR1 (Dharmacon, L-006526-00-0005) and nontargeting siRNA pool (Dharmacon, D-001810-10-20) were used at the final concentration of 10 nM. Knock-down efficiency was assessed after 72 hours through immunoblot and qPCR analysis.

### Colony-forming unit assay

1 × 10^3^ cells were plated into 2 mL of complete media in a 6-well plate format. In the case of treatments, media was replaced 48 hours after plating, and DMSO and Trametinib were added at a final concentration of 100 nM to 10 µM. After 9 days in culture, colonies were fixed in 4% PFA and stained using Toluidine Blue. Colonies were scored using ImageJ software.

### Three-dimensional spheroids formation and growth quantification

1 × 10^3^ cells were plated in ultralow attachment plates (96-well format, Perkin Elmer) in a final volume of 100 µL. After seeding, plates were centrifuged at 2,500*g* for 10 minutes at RT and incubated at 37 degrees with 5% CO_2_. After 24 hours from plating, the extracellular matrix (Geltrex, Thermo Fisher Scientific, cat. no. A1413201) was added in a 3% final concentration. Z-stack images of each spheroid were acquired daily starting from 48 hours using the Operetta CLS High Content Imager (Perkin Elmer). The area of spheroids (maximal projection) was quantified using a dedicated script developed with the Harmony software (Perkin Elmer).

### Wound healing assay

9 × 10^4^ HeLa FLCN KO cells engineered for TFEB expression were seeded in 70 µL of complete growth media in biocompatible silicone wells with a defined 500 μm cell-free gap (Culture-Inserts 2 well Cat. No: 80209; IBIDI) in a 24-well plate format and incubated at 37 degrees with 5% CO_2_. The next day, the silicon wells were removed and washed twice with complete growth media to prevent floating cells to attach to the gap. The images were acquired (every 30 minutes from the removal of the silicone wells for 24 hours) with the High Content Imager System Operetta CLS (PerkinElmer) with temperature (37°C) and CO_2_ (5%) controlled. The first and last acquisitions were used to perform the analysis (T = 0 and T = 24). Through the Harmony software (PerkinElmer) tool termed “*Measure distance/angle*,” 4 distances (between edges) were measured for each condition at indicated time points, and the distance covered by cell edges was quantified.

### RNA-sequencing library preparation and sequencing

Total RNA was quantified using the Qubit 2.0 fluorimetric Assay (Thermo Fisher Scientific). Libraries were prepared from 250 ng of total RNA using the 3′ DGE mRNA-seq sequencing service (TIGEM NGS Core & Next Generation Diagnostic Srl), which included library preparation, quality assessment, and sequencing on a NovaSeq 6000 sequencing system using a single-end, 100-cycle strategy (Illumina).

### RNA-sequencing data preprocessing and analysis

The raw data were analyzed by Next Generation Diagnostics Srl proprietary 3′ DGE mRNA-seq pipeline (v2.0), which involves a cleaning step by quality filtering and trimming, alignment to the reference genome, and counting by gene. Data were normalized via the *cpm* function from the edgeR package (v. 3.34.1). Differential expression analyses were performed using edgeR (v. 3.34.1) [47] on genes having more than 1 CPM in more than the minimum number of samples belonging to one condition minus 1 and less than 20% of multi-mapping reads, simultaneously. In general, genes were considered differentially expressed when displaying FDR < 0.05 and log_2_ fold change > 0 (up-regulated) or < 0 (down-regulated). To be more stringent, genes bound by EGR1 were considered deregulated with a fold change > 1.5 (UP) and < 1.5 (DOWN) when comparing 6h starvation with the fed control.

### Pathway and custom genesets enrichment analysis

Pathway enrichment analysis was conducted using the enrichR (v. 3.0) package [48–50] using KEGG [51] and MSigDB Hallmark collection [52] (Curated Pathways). Custom Genesets (Autophagy and Lysosome) enrichment analysis was performed using the *fisher*.*test* function from R (v. 4.2.0).

### Chromatin immunoprecipitation library preparation and sequencing

10^7^ cells were fixed with 1% formaldehyde for 15 minutes at RT. Samples for ChIP-seq were prepped as previously described [46]. Libraries were prepared from 10 ng of DNA using the NEBNext Ultra II DNA Library Prep Kit for Illumina (New England Biolabs). The quality of libraries was assessed using Bioanalyzer DNA Analysis (Agilent Technologies) and quantified using Qubit 4 Fluorometer (Thermo Fisher Scientific). Libraries were sequenced on a NovaSeq 6000 sequencing system using a paired-end (PE) 100 cycles flow cell (Illumina).

### ChIP-sequencing bioinformatic analysis

Paired sequencing reads were aligned on human GRCh38 reference genome using BWA[BWA] and filtered with samtools [samtools] to remove unmapped read pairs, not primary alignment, reads failing platform quality, with mapping quality score below 30, and duplicate reads were then removed using Picard MarkDuplicates [picard]. Each sample was equally split into 2 pseudoreplicates, peaks were called with MACS2[MACS] with *p* < 0.1 on both samples and pseudoreplicates and filtered after Irreproducible Discovery Rate analysis [IDR] with a threshold of 0.05. The coverage signal profile was generated with deep tools [deeptools] using CPM normalization.

## Supporting information

S1 Fig The architectural structure of human TFEB locus. **(A)** (Left) Boxplot showing the gene expression distribution of TFEB across different tissues. The average nTPM value for each tissue has been recovered from Human Protein Atlas (proteinatlas.org). (Right) Boxplot showing the gene expression distribution of TFEB across different cell lines. The CPM value for each cell line has been quantified from UCSC Transcription data (S1C Fig). (**B**) Structure of the human TFEB locus. Locus coordinates and encoded protein lengths of TFEB isoforms are indicated, along with coding exons (black boxes), UTR regions (red boxes), and directionality (arrows). The primary reference transcriptional isoform is underlined. (**C**) UCSC genome browser visualization of normalized RNAseq, H3K4me1, H3K4me3, and DNase profiles at the indicated locus in 6 ENCODE reference cell lines (colors). Arrow #1 indicates the transcriptional start site of the major TFEB isoform common to the reference cell lines. Arrow #2 corresponds to the region where most active H3K4me3 marks are located. Arrow #3 corresponds to the most accessible DNAseI region. Arrow #4 highlights the location of the chromatin loop enclosing the TFEB locus with likely carryover epigenetics and chromatin marks from the promoter region. (**D**) TFEB CREs are indicated on the bottom (from 1 to 7). The promoter region, which includes CREs 1, 2, and 3, is displayed in red. Chromatin folding profile (Hi-C HFF) is also displayed, reporting only the strongest interactions. Individual quantitative observations that underlie the data summarized here can be located under the Supporting information file as S1 Data. CRE, *cis*-regulatory element; TFEB, transcription factor EB.
(TIF)

S2 Fig EGR1 gain-of-function. **(A**) Balloon plots of representative term enrichment analysis results using Custom Genesets (Autophagy and Lysosome) and Curated Pathways (KEGG and MSigDB Hallmark collection) of genes up-regulated upon EGR1 overexpression, with respect to control cells (CTRL). Enriched terms are ranked by adjusted *p*-value (x-axis), and the balloon color scale represents the percentage of overlap between the input genes and the analyzed term. Significance threshold (dashed line, adjusted *p*-value < 0.05) is reported. (**B**) RNAseq-based expression (CPM) of representative genes upregulated upon CTRL and EGR1 overexpression in HeLa cells. Mean ± SD values are shown. Individual quantitative observations that underlie the data summarized here can be located under the Supporting information file as S1 Data. CTRL, control; EGR1, early growth response 1.
(TIF)

S3 Fig Genome-wide evaluation of EGR1 association on chromatin during starvation. **(A**) (Left) Schematic representation of EGR1 target genes, as defined by ChIPseq analysis, and their corresponding dynamics at 6 hours of starvation (UP, DOWN, and NDE). EGR1 targets were defined by evaluating their enrichment within 2 kb of the transcriptional start site of the corresponding genes. (Right) Pie chart showing the percentage of EGR1 target genes, which undergo transcriptional changes in starvation with respect to fed conditions. (**B**) Balloon plot of representative term enrichment analysis results using Curated Pathways (KEGG and MSigDB Hallmark collection) of EGR1 targets up-regulated in starvation. Enriched terms are ranked by adjusted *p*-value (x-axis), and the balloon color scale represents the percentage of overlap between the input genes and the analyzed term. Significance threshold (dashed line, adjusted *p*-value < 0.05) is reported. Individual quantitative observations that underlie the data summarized here can be located under the Supporting information file as S1 Data. ChIPseq, chromatin immunoprecipitation sequencing; EGR1, early growth response 1; NDE, not differentially expressed.
(TIF)

S4 Fig TFEB overexpression promotes cell migration. **(A**) Representative images of wound healing assay acquired at 24 hours from the removal of silicone insert. HeLa-FLCN KO expressing low (+TFEB low) or high (+TFEB high) levels of TFEB, along with control cells (CTRL) were employed for the migration assay. (**B**) Histogram of High-Content Imaging-based quantification of the distance between edges measured at time 0 (T0) and 24 hours (T24) after the removal of silicone insert relative to the cells indicate above. ANOVA followed by Tukey’s multiple comparisons test was used; *p < 0.05, **p < 0.01, ***p < 0.001. Individual quantitative observations that underlie the data summarized here can be located under the Supporting information file as S1 Data. CTRL, control; TFEB, transcription factor EB.
(TIF)

S5 Fig Transcriptomic analysis of HeLa-FLCN KO cells upon Trametinib treatment. **(A**) Bar plots showing relative quantification of EGR1 and TFEB mRNA levels measured by qPCR in HeLa cells treated with DMSO or Trametinib in fed conditions (FED) and after 8 hours of starvation (HBSS). Values were normalized on the HPRT expression and displayed as fold change relative to DMSO in FED. Mean ± SD values are shown. ANOVA was used; *p < 0.05, **p < 0.01, ***p < 0.001. (**B**) Immunoblot analysis of EGR1, TFEB, ERK, and pERK expression in HeLa cells treated with DMSO or Trametinib in fed condition (FED) and after 8 hours of starvation (HBSS). GAPDH was used as a loading control. (**C**) Volcano plot showing the results of the differential expression analysis in HeLa-FLCN KO cells upon Trametinib treatment compared to DMSO, as a function of log_2_ fold change (x-axis) and -log10 FDR (y-axis). Up- and down-regulated genes are highlighted (UP: 2,710 in red, DOWN: 2,712 in blue, NDE in gray). (**D**) Balloon plot of representative term enrichment analysis results using Curated Pathways (KEGG and MSigDB Hallmark collection) relative to down-regulated genes upon Trametinib treatment in HeLa-FLCN KO cells. Enriched terms are ranked by adjusted *p*-value (x-axis), and the balloon color scale represents the percentage of overlap between the input genes and the analyzed term. Significance threshold (dashed line, adjusted *p*-value < 0.05) is reported. Individual quantitative observations that underlie the data summarized here can be located under the Supporting information file as S1 Data. Uncropped images can be found in the Supporting information file as S1 Raw Images. EGR1, early growth response 1; NDE, not differentially expressed pERK, phospho-ERK; TFEB, transcription factor EB.
(TIF)

S1 Data Quantitative data underlying each figure (Figs 1–4 and S1–S5). Each tab contains the panels relative to the indicated figure for which a quantitative analysis was required.
(XLSX)

S1 Raw Images Raw images relative to the immune blots included in the manuscript. (ZIP)

## Abbreviations

BHD: Birt-Hogg-Dubé
CFU: colony formation assay
ChIPseq: chromatin immunoprecipitation sequencing
CRE: *cis*-regulatory element
CTRL: control
EGR1: early growth response 1
FLuc: firefly luciferase
GO: gene ontology
hESC: human embryonic stem cell
IEG: immediate-early gene
MAPK: mitogen-activated protein kinase
MOI: multiplicity of infection
shEGR1: short-hairpin RNAs against EGR1
TFEB: transcription factor EB
WT: wild-type
