# Supplementary figures and images for: EGR1 drives cell proliferation by directly stimulating TFEB transcription in response to starvation
Source: PLoS Biol. 2023 Mar 8;21(3):e3002034. doi: 10.1371/journal.pbio.3002034 (PMC9994711; doi:10.1371/journal.pbio.3002034)

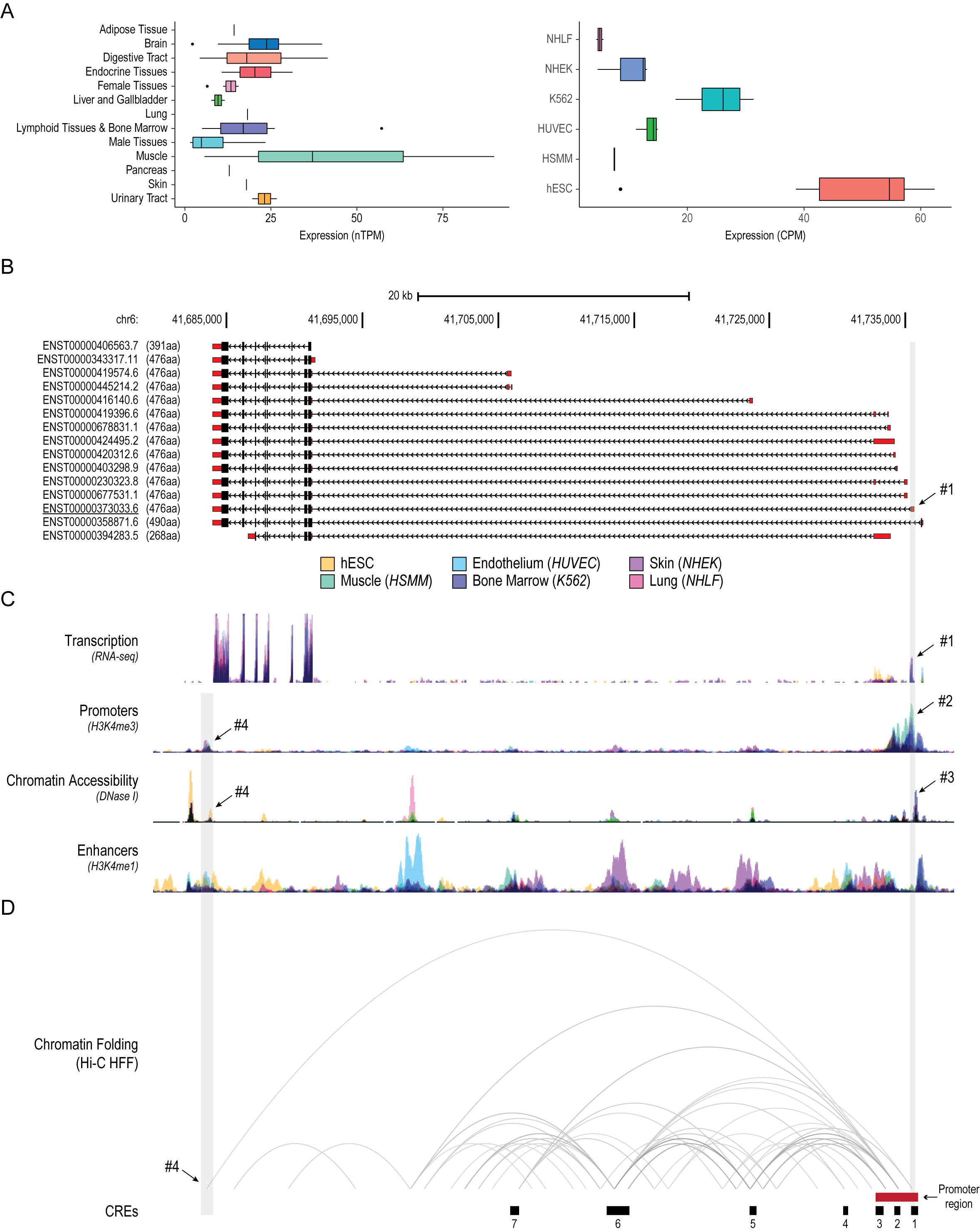

Supplement: S1 Fig — (A) (Left) Boxplot showing the gene expression distribution of TFEB across different tissues. The average nTPM value for each tissue has been recovered from Human Protein Atlas (proteinatlas.org). (Right) Boxplot showing the gene expression distribution of TFEB across different cell lines. The CPM value for each cell line has been quantified from UCSC Transcription data (S1C Fig). (B) Structure of the human TFEB locus. Locus coordinates and encoded protein lengths of TFEB isoforms are indicated, along with coding exons (black boxes), UTR regions (red boxes), and directionality (arrows). The primary reference transcriptional isoform is underlined. (C) UCSC genome browser visualization of normalized RNAseq, H3K4me1, H3K4me3, and DNase profiles at the indicated locus in 6 ENCODE reference cell lines (colors). Arrow #1 indicates the transcriptional start site of the major TFEB isoform common to the reference cell lines. Arrow #2 corresponds to the region where most active H3K4me3 marks are located. Arrow #3 corresponds to the most accessible DNAseI region. Arrow #4 highlights the location of the chromatin loop enclosing the TFEB locus with likely carryover epigenetics and chromatin marks from the promoter region. (D) TFEB CREs are indicated on the bottom (from 1 to 7). The promoter region, which includes CREs 1, 2, and 3, is displayed in red. Chromatin folding profile (Hi-C HFF) is also displayed, reporting only the strongest interactions. Individual quantitative observations that underlie the data summarized here can be located under the Supporting information file as S1 Data. CRE, cis-regulatory element; TFEB, transcription factor EB. (TIF) [file pbio.3002034.s001.tif]

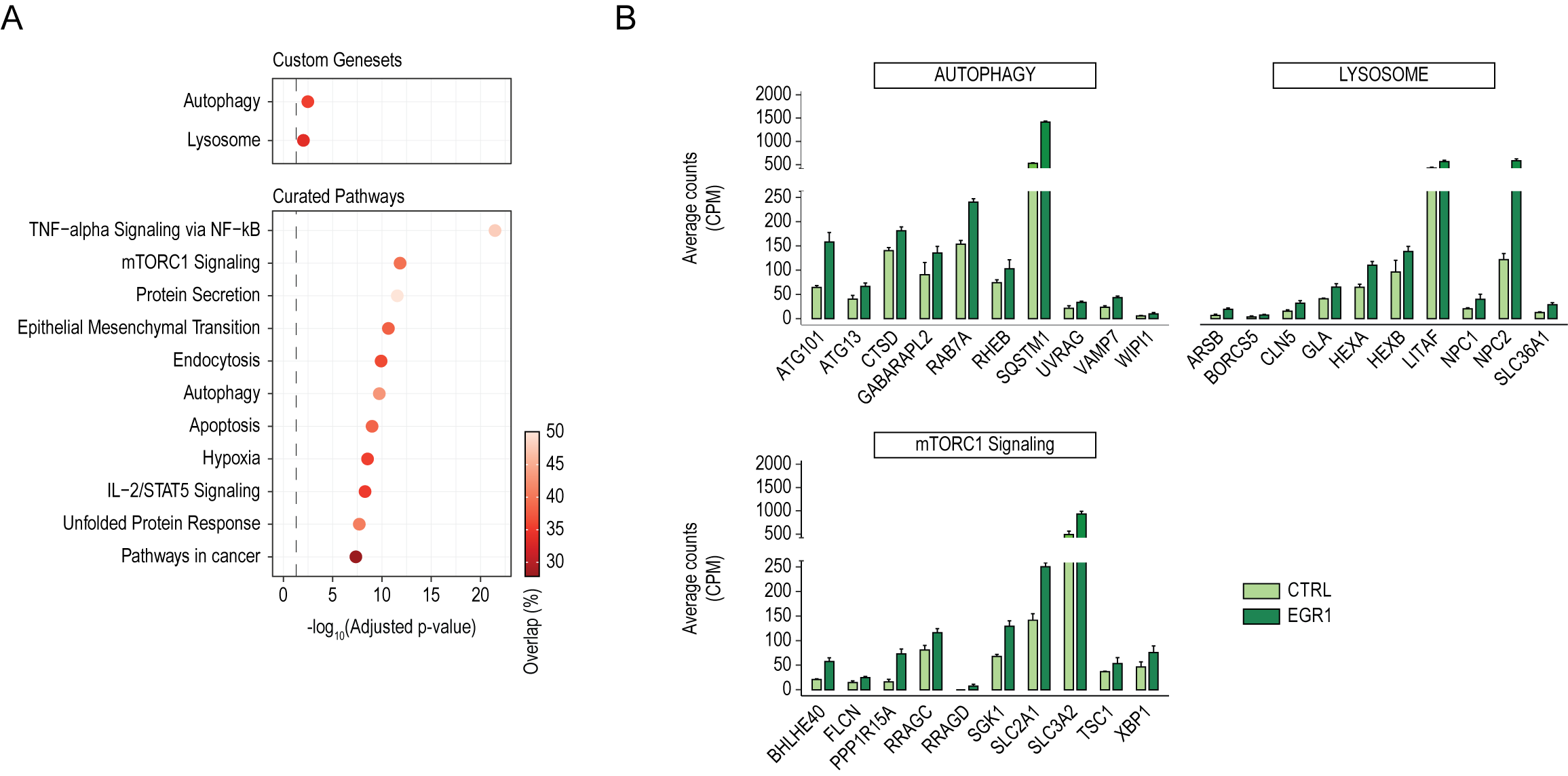

Supplement: S2 Fig — (A) Balloon plots of representative term enrichment analysis results using Custom Genesets (Autophagy and Lysosome) and Curated Pathways (KEGG and MSigDB Hallmark collection) of genes up-regulated upon EGR1 overexpression, with respect to control cells (CTRL). Enriched terms are ranked by adjusted p-value (x-axis), and the balloon color scale represents the percentage of overlap between the input genes and the analyzed term. Significance threshold (dashed line, adjusted p-value < 0.05) is reported. (B) RNAseq-based expression (CPM) of representative genes upregulated upon CTRL and EGR1 overexpression in HeLa cells. Mean ± SD values are shown. Individual quantitative observations that underlie the data summarized here can be located under the Supporting information file as S1 Data. CTRL, control; EGR1, early growth response 1. (TIF) [file pbio.3002034.s002.tif]

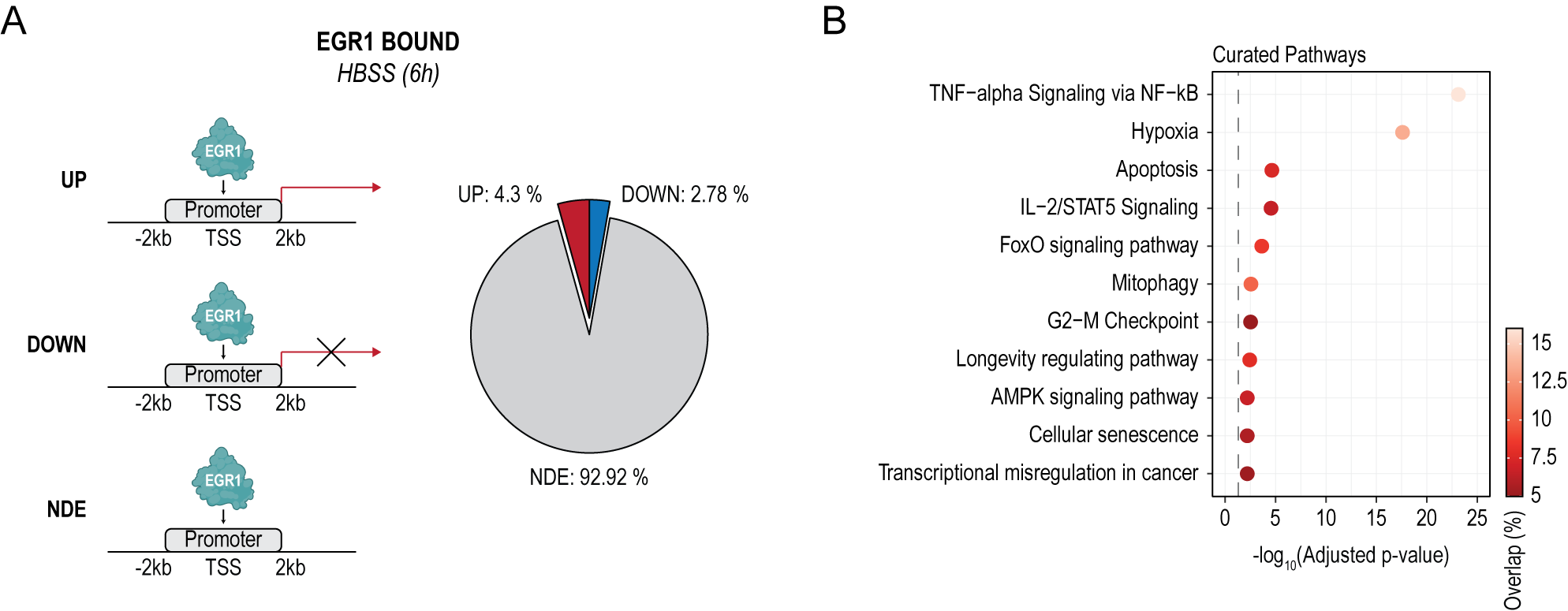

Supplement: S3 Fig — (A) (Left) Schematic representation of EGR1 target genes, as defined by ChIPseq analysis, and their corresponding dynamics at 6 hours of starvation (UP, DOWN, and NDE). EGR1 targets were defined by evaluating their enrichment within 2 kb of the transcriptional start site of the corresponding genes. (Right) Pie chart showing the percentage of EGR1 target genes, which undergo transcriptional changes in starvation with respect to fed conditions. (B) Balloon plot of representative term enrichment analysis results using Curated Pathways (KEGG and MSigDB Hallmark collection) of EGR1 targets up-regulated in starvation. Enriched terms are ranked by adjusted p-value (x-axis), and the balloon color scale represents the percentage of overlap between the input genes and the analyzed term. Significance threshold (dashed line, adjusted p-value < 0.05) is reported. Individual quantitative observations that underlie the data summarized here can be located under the Supporting information file as S1 Data. ChIPseq, chromatin immunoprecipitation sequencing; EGR1, early growth response 1; NDE, not differentially expressed. (TIF) [file pbio.3002034.s003.tif]

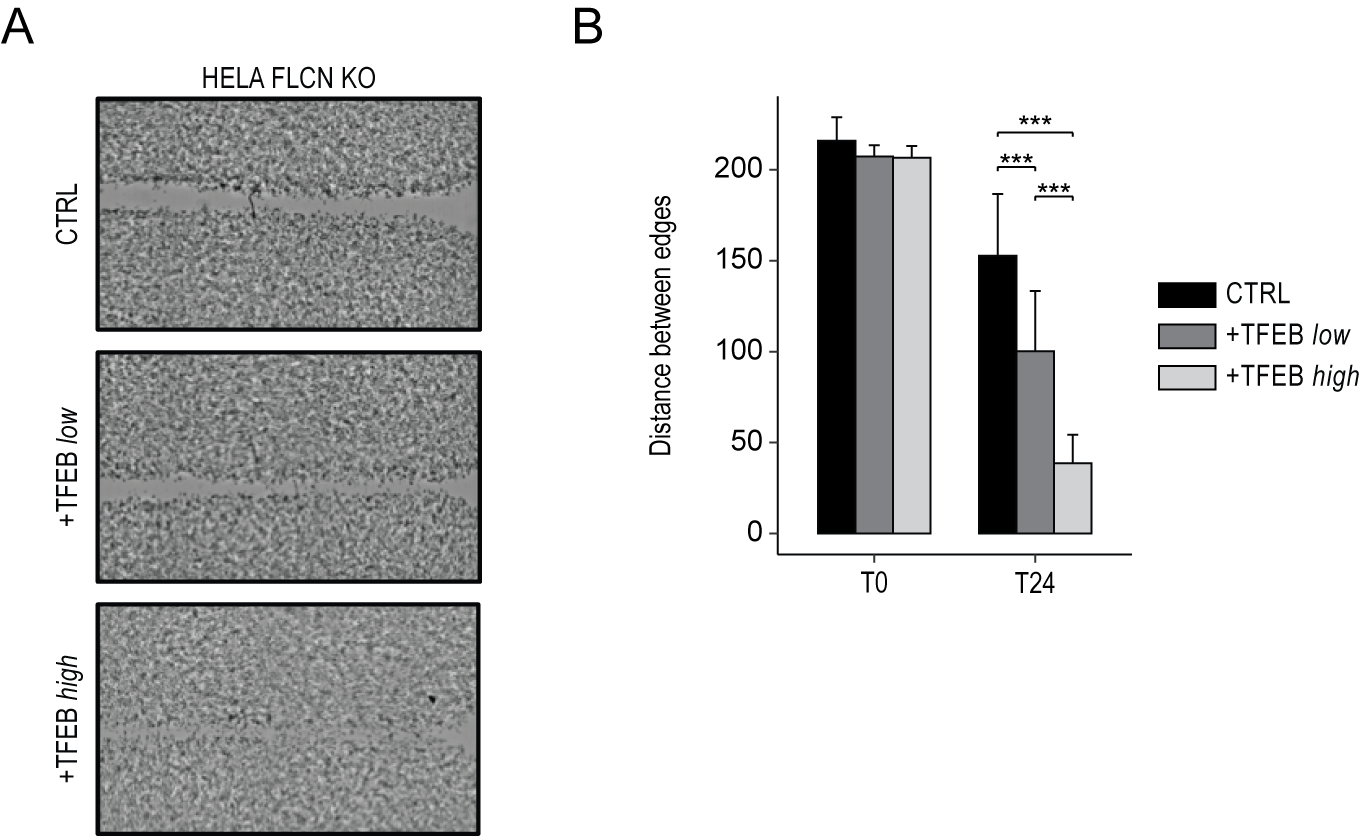

Supplement: S4 Fig — (A) Representative images of wound healing assay acquired at 24 hours from the removal of silicone insert. HeLa-FLCN KO expressing low (+TFEB low) or high (+TFEB high) levels of TFEB, along with control cells (CTRL) were employed for the migration assay. (B) Histogram of High-Content Imaging-based quantification of the distance between edges measured at time 0 (T0) and 24 hours (T24) after the removal of silicone insert relative to the cells indicate above. ANOVA followed by Tukey’s multiple comparisons test was used; *p < 0.05, **p < 0.01, ***p < 0.001. Individual quantitative observations that underlie the data summarized here can be located under the Supporting information file as S1 Data. CTRL, control; TFEB, transcription factor EB. (TIF) [file pbio.3002034.s004.tif]

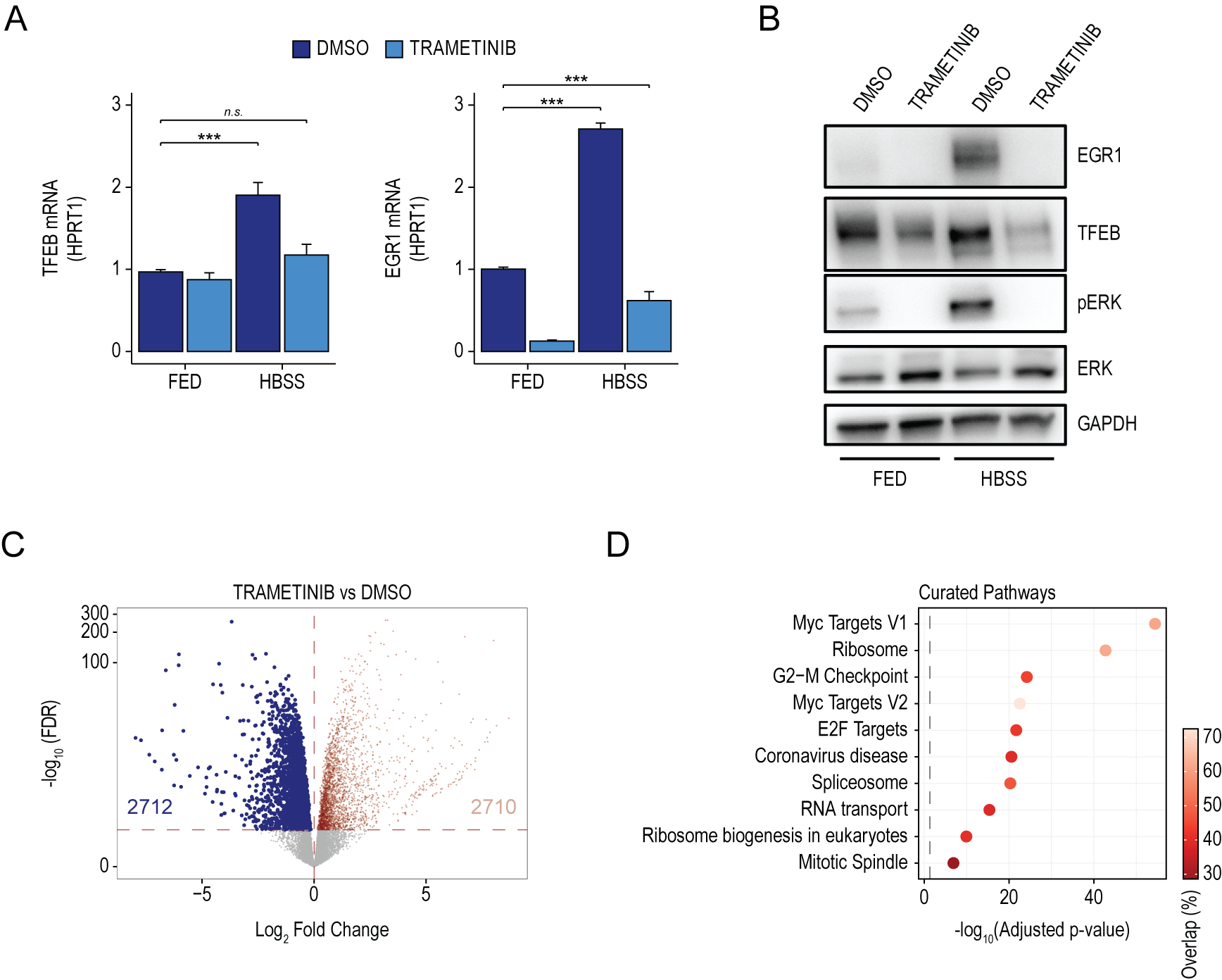

Supplement: S5 Fig — (A) Bar plots showing relative quantification of EGR1 and TFEB mRNA levels measured by qPCR in HeLa cells treated with DMSO or Trametinib in fed conditions (FED) and after 8 hours of starvation (HBSS). Values were normalized on the HPRT expression and displayed as fold change relative to DMSO in FED. Mean ± SD values are shown. ANOVA was used; *p < 0.05, **p < 0.01, ***p < 0.001. (B) Immunoblot analysis of EGR1, TFEB, ERK, and pERK expression in HeLa cells treated with DMSO or Trametinib in fed condition (FED) and after 8 hours of starvation (HBSS). GAPDH was used as a loading control. (C) Volcano plot showing the results of the differential expression analysis in HeLa-FLCN KO cells upon Trametinib treatment compared to DMSO, as a function of log2 fold change (x-axis) and -log10 FDR (y-axis). Up- and down-regulated genes are highlighted (UP: 2,710 in red, DOWN: 2,712 in blue, NDE in gray). (D) Balloon plot of representative term enrichment analysis results using Curated Pathways (KEGG and MSigDB Hallmark collection) relative to down-regulated genes upon Trametinib treatment in HeLa-FLCN KO cells. Enriched terms are ranked by adjusted p-value (x-axis), and the balloon color scale represents the percentage of overlap between the input genes and the analyzed term. Significance threshold (dashed line, adjusted p-value < 0.05) is reported. Individual quantitative observations that underlie the data summarized here can be located under the Supporting information file as S1 Data. Uncropped images can be found in the Supporting information file as S1 Raw Images. EGR1, early growth response 1; NDE, not differentially expressed pERK, phospho-ERK; TFEB, transcription factor EB. (TIF) [file pbio.3002034.s005.tif]

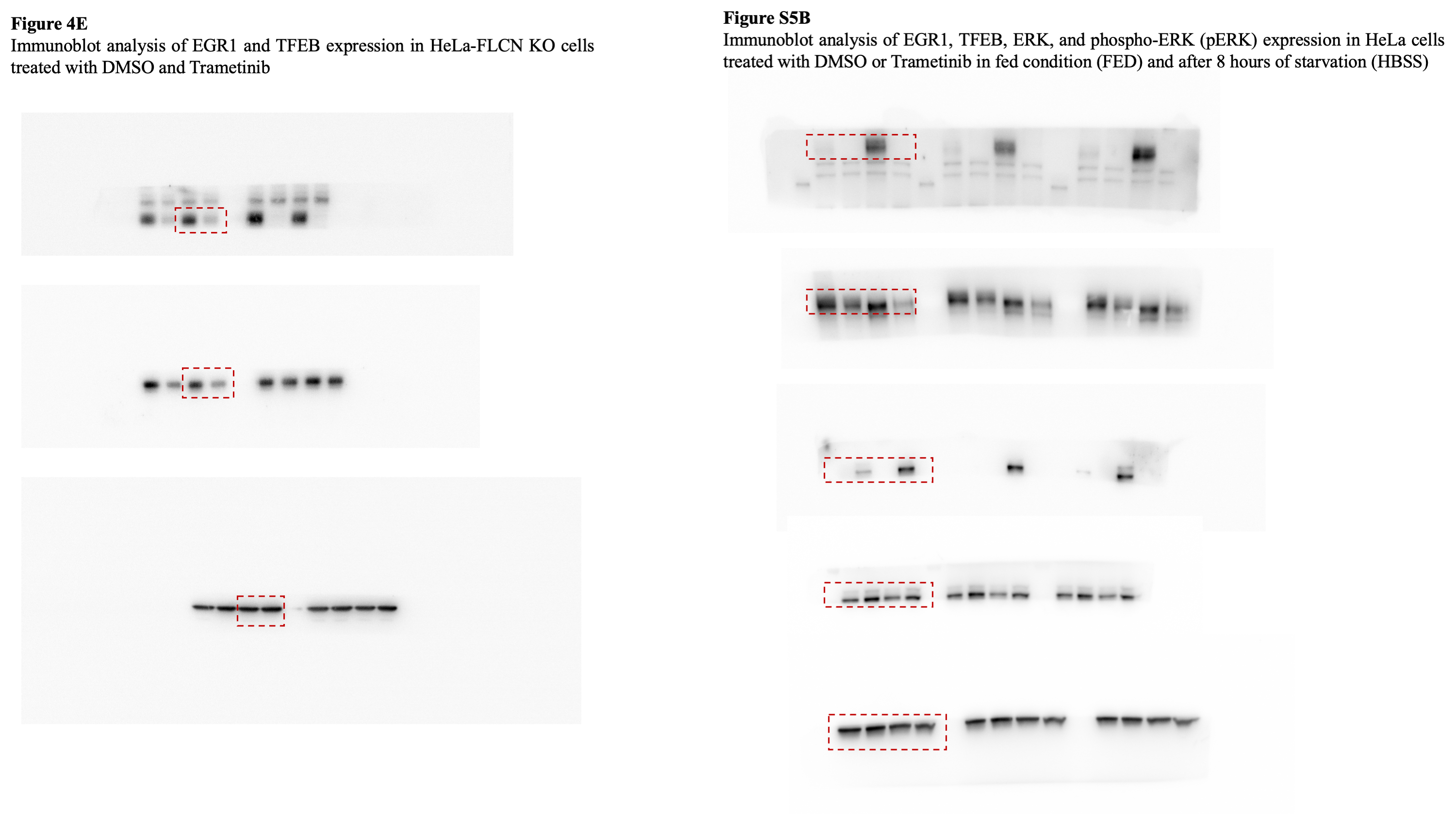

Supplement: S1 Raw Images — (ZIP) [file pbio.3002034.s007.zip › S1_Raw images/Slide3.tiff]

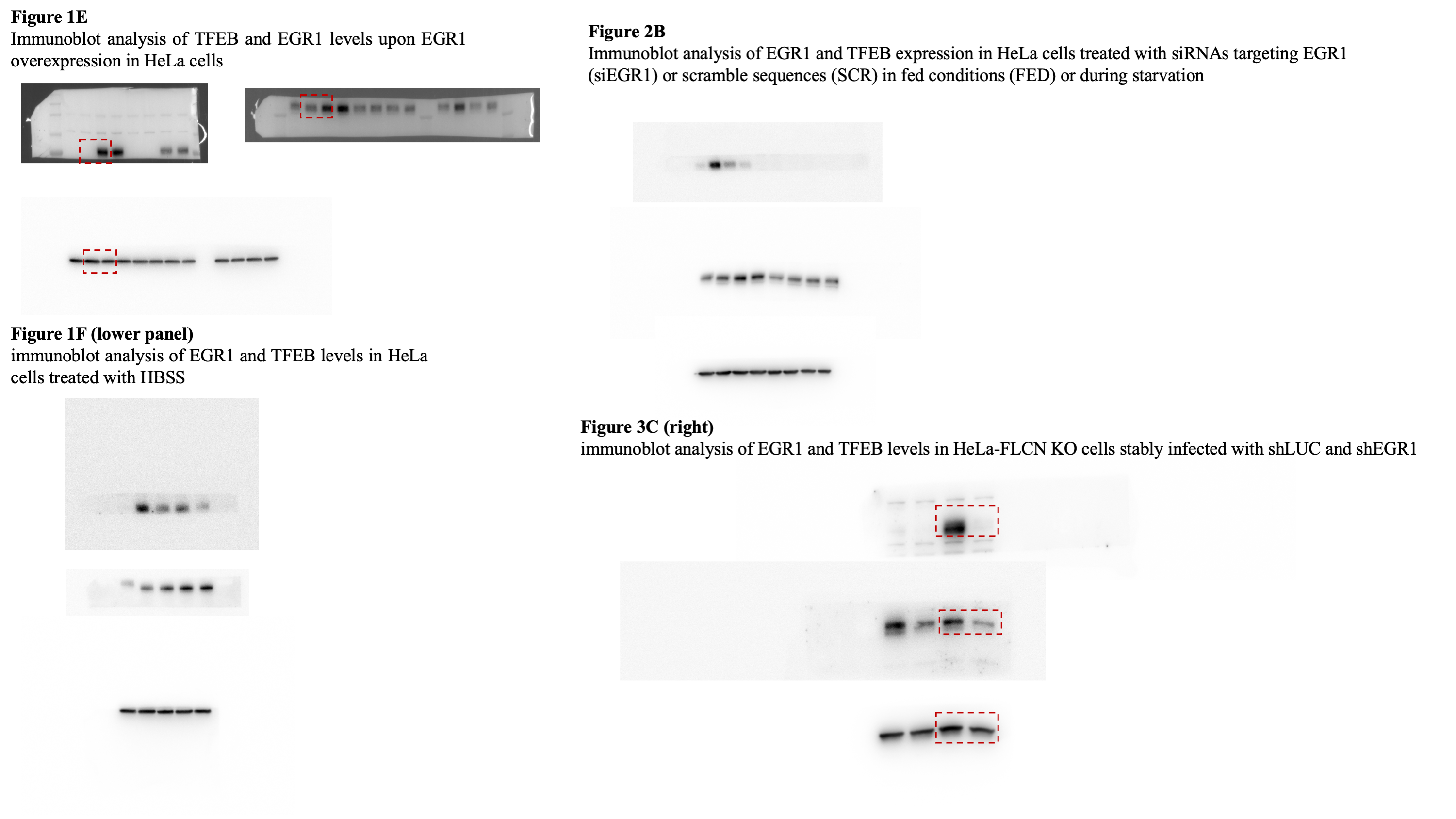

Supplement: S1 Raw Images — (ZIP) [file pbio.3002034.s007.zip › S1_Raw images/Slide2.tiff]
